# Cutting carbon and nitrogen footprints of maize production by optimizing nitrogen management under different irrigation methods

**DOI:** 10.3389/fpls.2024.1476710

**Published:** 2024-12-04

**Authors:** Yunfei Di, Yu Gao, Haibo Yang, Dong Yan, Yuzhe Tang, Weijian Zhang, Yuncai Hu, Fei Li

**Affiliations:** ^1^ College of Resources and Environmental Sciences, Inner Mongolia Agricultural University, Hohhot, China; ^2^ Inner Mongolia Key Laboratory of Soil Quality and Nutrient Resources, Key Laboratory of Agricultural Ecological Security and Green Development at Universities of Inner Mongolia Autonomous Region, Hohhot, China; ^3^ Department of Soil Fertilizer and Water Saving Agricultural Technology, Inner Mongolia Agriculture and Animal Husbandry Technology Popularization Center, Hohhot, China; ^4^ Department Life Science Engineering, School of Life Sciences, Technical University of Munich, Freising, Germany

**Keywords:** carbon and nitrogen footprints, nitrogen management, greenhouse gas emission, reactive nitrogen losses, life cycle assessment

## Abstract

**Introduction:**

Analyzing the effects of nitrogen (N) fertilizer application and water management on the carbon (C) and N footprints is vital to maize production systems.

**Methods:**

This study conducted field experiments from 2019-2020 involving flood- and drip-irrigated maize production systems in Northwest China to analyze N and C footprints (NF and CF, respectively) based on the life cycle assessment (LCA). The N fertilizer treatments studied included no N fertilizer application (Control), optimized N management (OM), optimized N management incorporated with urease inhibitor (OMI, UI), and farmer practice (FP).

**Results and discussion:**

The maize grain yields under flood irrigation afforded by OMI (12.3 t ha^-1^) and FP treatments (13.4 t ha^-1^) were significantly higher than that of OM treatment (11.0 t ha^-1^). But maize grain yields of the OM (12.1 t ha^-1^), OMI (12.5 t ha^-1^), and FP treatments (12.5 t ha^-1^) showed no significant difference under drip irrigation although less N was applied to OM and OMI. The OMI treatment had better environmental effects than the OM treatment under both flood and drip irrigation. Applying N fertilizer with UI increased N use efficiency (NUE) and reduced N losses under flood irrigation. The reactive N (Nr) losses, greenhouse gas (GHG) emissions, NF, and CF of OMI treatment were 43.9%, 45.3%, 35.7%, and 37.4% lower under flood irrigation (77.6 kg N ha^-1^, 4499.9 kg CO2 eq ha^-1^, 6.7 kg N t^-1^, and 387.7 CO2 eq N t^-1^) and 43.3%, 37.1%, 43.2%, and 37.1% lower under drip irrigation (57.8 kg N ha^-1^, 4144.3 kg CO2 eq ha^-1^, 4.7 kg N t^-1^, and 332.7 CO2 eq N t^-1^) compared to the FP treatment. The Nr losses, GHG emissions, NF, and CF of drip irrigation were lower than those of flood irrigation. According to the analysis of driven indicators, the N leaching, electricity for irrigation, and NH_3_ volatilization were the most important contributors to the NF; the fertilizer, electricity for irrigation, and N_2_O emissions were the dominant factors controlling the CF. The environmental impact of the OMI treatment was less than that of the OM and FP treatments. Therefore, integrating better N management practices and efficient irrigation methods can significantly reduce environmental impacts while maintaining yields in maize cultivation.

## 1 Introduction

Nitrogen (N) is the most important crop nutrient for achieving optimum crop yields (Cui et al., 2018). However, N fertilizer application in high-yielding cropping systems has caused severe environmental problems (Li et al., 2020; Sun et al., 2022). Excessive N fertilization is common and causes enormous N losses in intensive irrigation-based agricultural production regions in China, such as the North China Plain and the Northeast Plain (Chen et al., 2014; Shi et al., 2020). A large amount of inevitable N losses are associated with low N use efficiency (NUE) and more N in runoff of surface water, N leaching into the ground water, production of atmospheric nitrous oxide (N_2_O) and ammonia (NH_3_) emission into the air, and reduced biodiversity (Kim et al., 2022; Xia et al., 2016; Yang et al., 2020). Additionally, the excessive N fertilizer application also induces PM_2.5_ pollution and climate change effects due to high-level NH_3_ volatilization and N_2_O emissions (Shen et al., 2022). Therefore, the N management practices of many crop systems are developed to address the dual challenges of excessive fertilization and environmental impacts (Si et al., 2020; Zhang et al., 2017). However, few studies have considered the management conditions and crop types to optimize N and water management based on planting environments in the agro-pastoral ecotone of China.

In addition to N management, the method of irrigation is another important factor affecting crop growth and yield formation (Xu et al., 2019). The different distribution of global water resources presents a more prominent constraint on regional agricultural development due to water shortage (Yin et al., 2020). The FAO reported that over 60% of all irrigated areas face severe water shortages (FAO, 2020), especially in Northwest China (Yu et al., 2017), Africa (Nyam et al., 2021), and the Middle East (Wehbe and Temimi, 2021). As a result, agriculture has intensified to ensure food supplies in developing countries with large populations (Fischer and Connor, 2018; Hashemi et al., 2019). Many studies on developing agricultural water regimes and irrigation methods have emerged with the increase in global warming and irrigation requirements (Du et al., 2019; Liu et al., 2018). These studies indicated that optimizing the duration and frequency of irrigation significantly increased crop yield and water use efficiency (Huang et al., 2022). In addition, water conservation by using drip and sprinkler irrigation has been developed to address the challenges of water shortage and high irrigation frequency in arid and semi-arid regions (Sui et al., 2018; Wang et al., 2021). Therefore, most studies developed effective water regimes based on flood, furrow, drip, and sprinkler irrigation methods (Carrijo et al., 2017; Guo et al., 2021). However, integrating regional-specific N management practices with more efficient irrigation systems is urgently needed to achieve high crop yields while minimizing water use and environmental impacts.

The reactive N (Nr) losses and greenhouse gas (GHG) emissions of agriculture production input and output materials associated with a cropping system based on a life cycle assessment (LCA) methodology have generated increasing attention (ISO, 2006; Jiang et al., 2022; Zhou et al., 2019). The N and carbon (C) footprints (the quantity of total Nr losses and GHG emissions per ton of a standard crop yield) are widely used to evaluate the environmental impacts of agricultural production (Knudsen et al., 2014; Wang et al., 2020). Quantifying and analyzing the N and C footprints are essential for developing sustainable agriculture (Dachraoui and Sombrero, 2020; Zhang et al., 2018). Many studies have documented the effects of N management approaches or flood irrigation regimes on N and C footprints and how to mitigate the environmental impacts of cropping systems (Dachraoui and Sombrero, 2020; Jiang et al., 2019). These studies indicated that optimal N fertilization rates can effectively decrease the environmental footprints in flood- or furrow-irrigated cropping systems (Cui et al., 2018; Jiang et al., 2022). However, the characteristics of N and C footprints under combined region-specific N management and drip irrigation are still unclear. In addition, urease inhibitors (UI) increase NUE and reduce N losses by matching crop N demand with N input in modern agricultural systems (Cheng et al., 2022; Yao et al., 2021; Zhang et al., 2019). This effect is achieved by delaying urea hydrolysis and bacterial ammonium oxidation. The effectiveness of UI varies by soil properties, climate, crop types, and management practices (Elrys et al., 2021; Shi et al., 2017). Therefore, further exploration of UI use under region-specific and drip irrigation conditions would play a vital role in promoting sustainable agriculture.

Maize is the principal grain crop in arid and semi-arid regions of Northwest China that can be used to ensure the safety of grain production capacity (Guo and Liu, 2021). The region has a temperate continental climate with approximately 200–400 mm of average annual precipitation and over 2,000 mm of average yearly evaporation, making it suitable for crop growth with supplementary irrigation. The high-intensification maize monoculture system in the region is managed by smallholder farmers under flood irrigation and is characterized by excessive N fertilizer inputs, low NUE, and high environmental risks (Meng et al., 2018; Wei et al., 2020). The continuous monoculture and unreasonable irrigation regimes have severely affected environmental quality and surface- and ground-water levels in Northwest China. The local government has promoted drip irrigation to improve N management measures and conserve water resources. However, many farmers still use flood or furrow irrigation, which has high ecological costs and restricts sustainable development (Meng et al., 2018; Wei et al., 2020). Therefore, integrating N management using UI and drip irrigation for maize production may reduce environmental impact while maintaining crop yields.

Inappropriate N management approach, less efficient irrigation methods (flood or furrow irrigation), and declining ground water levels limit the sustainability of maize production in arid and semi-arid areas. The LCA has been widely used to analyze driven indicators and identify environmental footprints for sustainable agriculture associated with various cropping systems (Dachraoui and Sombrero, 2020). This project comprehensively evaluates the environmental impacts of achieving high-yield and efficient maize production between flood and drip irrigation based on LCA. The objectives of this 2-year field experiment with four N treatments under flood and drip irrigation in Northwest China were to 1) quantify the N and C emissions and footprints and determine the dominant contributing indicator; 2) evaluate the effectiveness of UI on improving NUE; and 3) explore the opportunities for alleviating environmental impacts of maize production under drip irrigation. The results of this study can be used to provide a basis for reducing environmental footprints and achieving sustainable agricultural production under drip irrigation.

## 2 Materials and methods

### 2.1 Site description and experimental design

The field experiments were conducted in 2019 and 2020 on maize cultivated under flood and drip irrigation with water from the Yellow River in Wuyuan County, Inner Mongolia (Wuyuan; 41°4′N, 108°2′E), China. The experimental site is located in a temperate, arid, and semi-arid continental climate. The annual temperature was 6.1°C and 6.3°C; precipitation was 173 mm and 171 mm; and the frost-free period was 117 and 126 days of the field experiments for 2019 and 2020, respectively. The main soil properties of the 0–30-cm layer before the experiment commenced were as follows: pH, 8.8; organic matter content, 7.4 g kg^−1^; nitrate-N content, 11.8 mg kg^−1^; olsen-phosphorus (P) content, 20.1 mg kg^−1^; and available potassium (K) content, 132.9 mg kg^−1^.

The field experiments were established with a randomized block design with a plot size of 6.5 m× 10 m. Four N treatments with four replications were employed: control (no N fertilizer application), OM (the optimized N management), OMI (optimized N management incorporated with 0.05% urease inhibitor), and FP (farmer practice). The N rate of the OM treatment was determined based on the maize production studies conducted by our group at the same location in previous years. The N rate of the FP treatment was based on broad surveys of the local farmers’ common practices in the region. The maize growth stages were based on the Biologische Bundesanstalt, Bundessortenamt, and CHemical industry (BBCH) classification (Lancashire et al., 1991). The N fertilizer was applied before sowing (30%), BBCH 30–39 (30%), BBCH 51–59 (30%), and BBCH 61–69 (10%), except for the Control treatment. Details concerning N fertilizer rates (urea 46% N) and maize cultivars in the two growing seasons are presented in **Table 1**. The maize variety was Xinyu 12 in 2019 and Jindan 42 in 2020 with a population of 75,000 plants ha^−1^. The calcium superphosphate (90 kg P_2_O_5_ ha^−1^) and potassium sulfate (120 kg K_2_O ha^−1^) were applied before sowing as basal fertilizers. The flood irrigation rates applied at specific growth stages were 120 mm at BBCH 30–39 and 90 mm at each of BBCH 51–59, BBCH 61–69, and BBCH 71–79. The drip irrigation rates applied at specific growth stages were 50 mm at each of BBCH 30–39, BBCH 51–59, BBCH 61–69, and 60 mm at both BBCH 71–79 and BBCH 83–89. The experiment’s tillage, herbicide, pesticide use, and other practices were the same as local farmers, except for N fertilizer application, irrigation, and maize harvesting.

**Table 1:** The rate of N fertilizer applied and split to different N treatments of maize production in 2019 (Xinyu 12) and 2020 (Jindan 42).

Treatment	N fertilizer rate (kg N ha^−1^)	Base fertilizer (kg N ha^−1^)	BBCH 30–39 (kg N ha^−1^)	BBCH 51–59 (kg N ha^−1^)	BBCH 61–69 (kg N ha^−1^)
Flood irrigation					
Control	0	0	0	0	0
OM	180	54	54	54	18
OMI	180	54	54	54	18
FP	400	120	120	120	40
Drip irrigation					
Control	0	0	0	0	0
OM	180	54	54	54	18
OMI	180	54	54	54	18
FP	400	120	120	120	40

Control, OM, OMI, FP, and BBCH represent i) no fertilizer N application, ii) optimize N management, iii) optimize N management incorporated with 0.05% urease inhibitor, iv) farmer practice, and v) Biologische Bundesanstalt, Bundessortenamt and CHemical industry, respectively.

**Figure 1:**
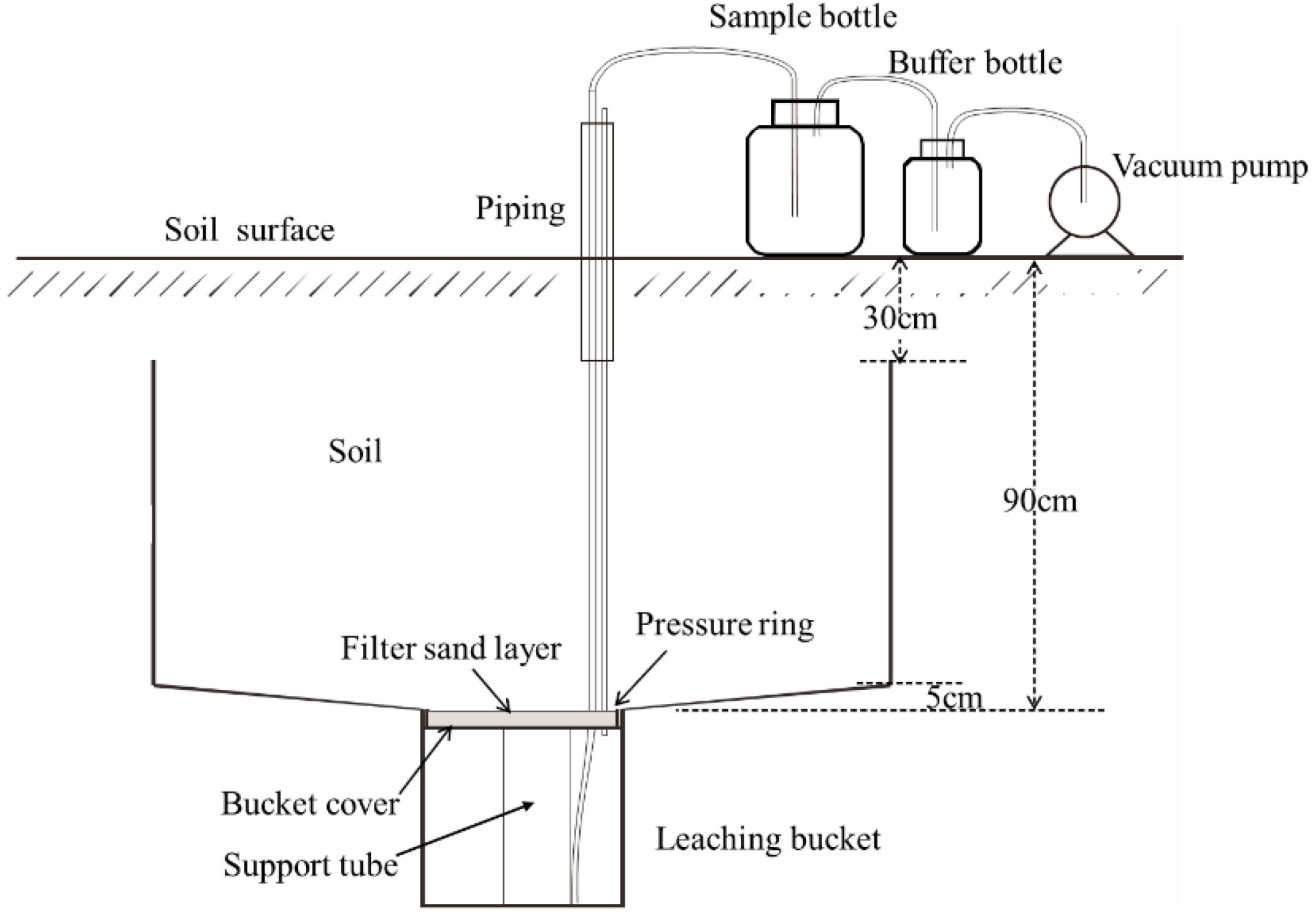
Schematic diagram of leachate collection device with plastic boundaries.

The maize plants covering an area of 6.6 m^2^ were manually harvested at maturity in the middle of each field plot to calculate yield. The N leaching, NH_3_ volatilization, and N_2_O emission were measured using the field percolation pond, glycerol phosphate-sponge ventilation method, and static chamber methods in the actual field experiments, respectively (Cui et al., 2024; Guo et al., 2022; Huang et al., 2017). The percolation water collection device is shown in **Figure 1**, which was embedded in a pit of 90 cm depth with a plastic boundary. The soil was excavated in a 30-cm increment layer and then returned to its original depth after installing the leaching bucket. The leachate was collected using an electrical vacuum pump of 100 kPa to a triangular flask during the maize growing stages (before sowing, BBCH 30–39, BBCH 51–59, BBCH 61–69, and BBCH 71–79 under flood irrigation and BBCH 30–39, BBCH 51–59, BBCH 61–69, BBCH 71–79, and BBCH 83–89 under drip irrigation). The leachate samples were stored in a 200-mL polyethylene bottle and immediately frozen at −20°C prior to analysis. The NH_3_ was measured by sponge tracking and the KCl extraction method *in situ* for the maize field (Cui et al., 2024). Two sponges (2 cm thick and 16 cm inner diameter) spiked with 15 mL of a glycerol-phosphoric acid (H_3_PO_4_, 85.0%) mixture with 40 mL of glycerol, 50 mL of H_3_PO_4_, and 910 mL of deionized water and then inserted into containers 10 cm long and 15 cm in inner diameter in experimental plots. One sponge was inserted 5 cm above the soil surface of the container and was used to trap the NH_3_ volatilized from the maize field. The other sponge was fitted into the top of the container to avoid contamination by atmospheric NH_3_. The NH_3_ trapped in the lower sponge was extracted with 1 M KCl. The N_2_O was collected between 08:30 and 11:30 on the morning of each sampling day by a stainless static chamber with a pedestal (50 cm long, 50 cm wide, and 70 cm high) during the maize growing season (Guo et al., 2022). The N_2_O samples were obtained using a 100-mL plastic tight syringe at 0 min, 10 min, 20 min, and 30 min after chamber closure and transferred to sealable airbags. The NH_3_ and N_2_O samplings were made at 15–25-day intervals, and continuous measurements lasted for 6 days with a frequency of 2 days after fertilization and irrigation during the whole maize growing season. The leachate and NH_3_ samples were analyzed by a continuous flow analyzer (TRAACS2000 system, Norderstedt, Germany), and N_2_O samples were analyzed by a gas chromatograph (Picarro G2308, Shanghai, China).

### 2.2 Life cycle inventory analysis and evaluation

**Figure 2:**
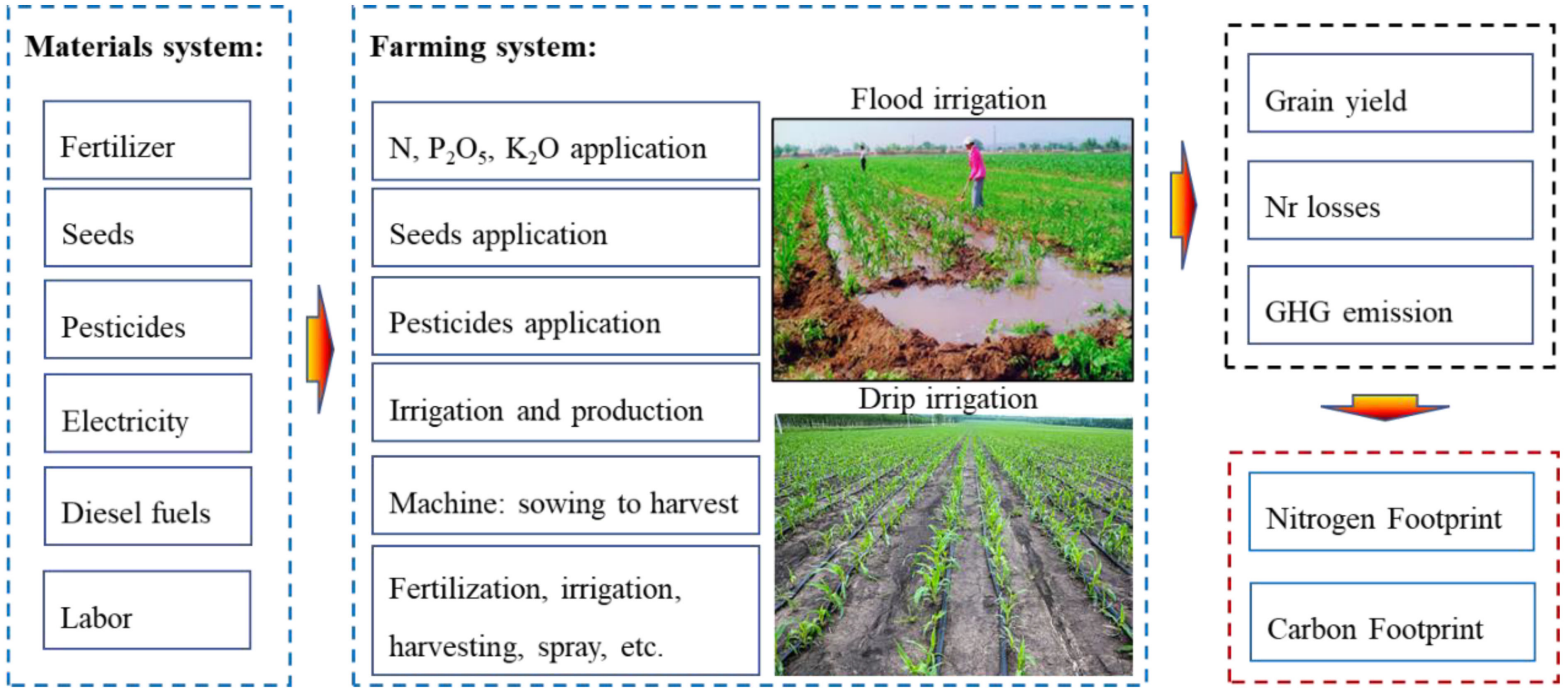
System boundaries and inventory adopted for life cycle assessment (LCA) of maize system.

The life cycle inventories include the production, transportation, and application of agricultural material inputs and outputs from the perspective of an LCA (ISO, 2006; Zhang et al., 2021). The Nr losses and GHG emissions of the entire growing period for the maize system were quantified from sowing to harvest based on LCA. The system boundary is shown in **Figure 2**.

#### 2.2.1 Reactive N losses and N footprint

The system boundary shows that the reactive N (Nr) losses included fertilizers (N, P_2_O_5_, and K_2_O), pesticides, seed, electricity for irrigation, diesel fuel consumption, labor, NH_3_–N volatilization, N_2_O–N emission, and N leaching of agricultural material production, transportation, and application. These were calculated using the following equations (Huang et al., 2021; Wang et al., 2020):


(1) $$\text{Nr}=\text{Nr}-\text{Mi}+\text{N leaching}+{\text{NH}}_{3}-\text{N}+{\text{N}}_{2}\text{O}-\text{N}$$

(2) Nr−Mi=∑(Ni×Ei)

(3) NF=Nr/maize yield

where Nr-Mi is the total Nr losses from fertilizers, pesticides, seed, electricity for irrigation, diesel consumption, and labor of agricultural materials production, transportation, and application. Nitrogen leaching, NH_3_–N, and N_2_O–N represent the rate of percolation loss from farmland, NH_3_ volatilization, and N_2_O emission, respectively. The Nr losses emission factor from each agricultural material was referenced in previous studies (Huang et al., 2021; Zhang et al., 2021). Ni represents the rate of the ith agricultural input material, Ei represents the emission factor of the ith agricultural input material, and i represents the various items of agricultural input materials. The Ei of agricultural materials are shown in **Table 2**. The NF represents the total Nr emission per ton of standard yield of the maize system.

**Table 2:** Emission factor of each agricultural material in the maize production system.

Items	Unit	Nr (kg N unit^−1^)	GHG (kg CO_2_ eq unit^−1^)	Reference
N	kg N	0.0075	8.3	Huang et al., 2021; Ling et al., 2021
P_2_O_5_	kg P_2_O_5_	0.00183	2.33	Liu et al., 2013; Huang et al., 2021; Ling et al., 2021
K_2_O	kg K_2_O	0.00146	0.66	Liu et al., 2013; Huang et al., 2021; Ling et al., 2021
Pesticides	kg	0.0469	19.13	Liu et al., 2013; Huang et al., 2021; Ling et al., 2021
Seed	kg	–	1.22	Liu et al., 2013
Diesel fuel	L	0.0286	3.75	Liu et al., 2013; Zhang et al., 2013; Wang et al., 2020
Electricity	kWh	0.0197	1.14	Liu et al., 2013; Zhang et al., 2013; Wang et al., 2020
Labor	person	–	0.86	Liu et al., 2013; Huang et al., 2021

#### 2.2.3 Greenhouse gas emissions and C footprint

The GHG emissions were calculated by the agricultural materials production, transportation, and application (GHG-Mi), N_2_O–N direct emission (N_2_O–N_direct_), and indirect (N_2_O–N_indirect_) emission. The N_2_O–N _indirect_ emission was calculated by the 1% and 1.1% emissions factors from NH_3_–N and N leaching, respectively (Huang et al., 2021; IPCC, 2019). The relationships are in the following equations:


(4) $$\text{GHG}=\text{GHG}-\text{Mi}+{\text{total N}}_{2}\text{O}-\text{N}\times \left(44/28\right)\times 298$$

(5) GHG−Mi=∑(Gi×Ei)

(6) $$\begin{array}{c} {\text{Total N}}_{2}\text{O}-\text{N}={\text{N}}_{2}\text{O}-{\text{N}}_{\text{direct}}+\;1\%\;\times {\text{ NH}}_{3}-\text{N}+1.1\%\;\\ \times \text{ N leaching} \end{array}$$

(7) C footprint=GHG/maize yield

where GHG-Mi is the GHG emissions from fertilizers, pesticides, seed, electricity for irrigation, diesel consumption, and labor of various agricultural materials production, transportation, and application. The total N_2_O–N represents the sum of the direct and indirect N_2_O–N emissions, 44/28 is the molecular weight ratio of N_2_O to N, 298 is the equivalent coefficient of N_2_O emissions for global warming potential (kg CO_2_ eq kg^−1^), Gi is the quantity of the ith individual agricultural input material, and EFi is the GHG emission factor of the ith agricultural input material, where i represents the various item of agricultural input materials. The values of each Ei are shown in **Table 2**. The CF was calculated as GHG emissions per ton of standard yield of maize.

### 2.3 Statistical analysis

A one-way variance analysis (ANOVA) of different treatments was performed using the least significant difference (LSD) test with the SPSS software (26.0 version). Tables and figures were constructed using Excel 2021 (Microsoft Corp., USA) and Origin 2024 (OriginLab, Corp., USA). The date values were means ± standard deviation (SD).

## 3 Results

### 3.1 Grain yield and economic benefits

**Figure 3:**
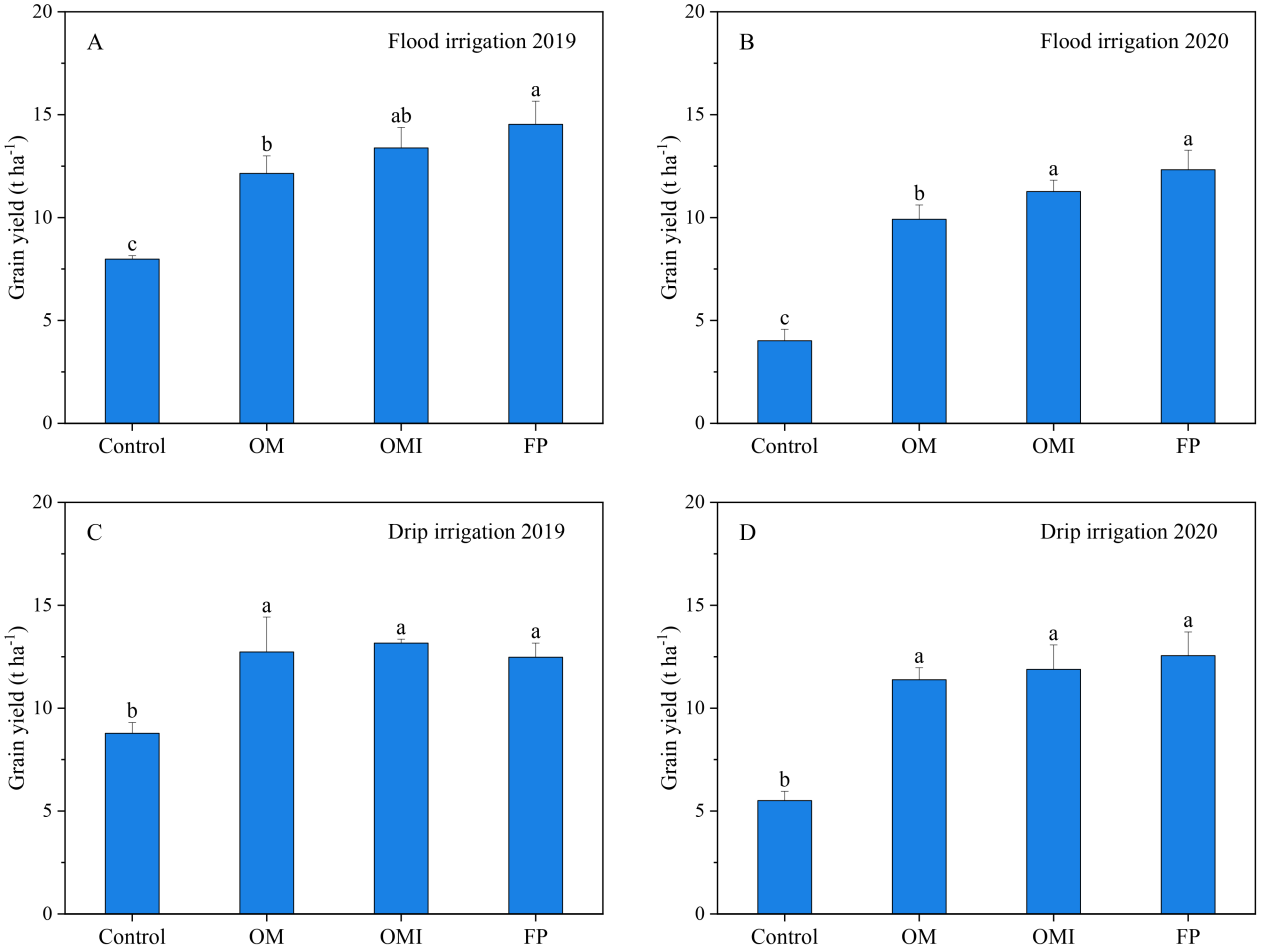
Grain yield in flood and drip irrigation of maize system. Panels **(A, B)** represent the annual grain yield of the flood irrigation system. Panels **(C, D)** represent the annual grain yield of the drip irrigation system. Different lowercase letters indicate significant differences at *p* < 0.05 (LSD).

Both N management and irrigation method directly influenced the maize grain yield (**Figure 3**). The OM and OMI considerably reduced the N fertilizer rate by 55.0% without compromising grain yield during the 2-year experiments compared to the FP treatment. The maize grain yield of OMI treatment was higher than that of OM treatment; thus, 10.2% (13.4 vs. 12.1 t ha^−1^) in 2019 and 13.6% (11.3 vs. 9.9 t ha^−1^) in 2020 more under flood and 3.4% (13.2 vs. 12.7 t ha^−1^) in 2019 and 4.4% (11.9 vs. 11.4 t ha^−1^) in 2020 under drip irrigation, respectively. The mean maize yield of OMI (12.3 t ha^−1^) treatment was 8.2% lower than that of FP (13.4 t ha^−1^) treatment under flood irrigation but similar to that of FP under drip irrigation (12.5 vs. 12.5 t ha^−1^). Compared to OMI and FP, the OM yield was significantly lower under flood but not drip irrigation (**Figure 3**). The OMI and FP yields did not differ significantly. The highest maize yield was recorded in 2019 for FP (14.5 t ha^−1^) treatment under flood irrigation and OMI (13.2 t ha^−1^) treatment under drip irrigation. The cost and benefits of input and output materials [fertilizers, drip tapes, irrigation, and others (seed, fuel for machinery, labor, and pesticides)] for maize production were calculated using the field experiments dataset (**Table 3**). As shown in the table, the benefits of OMI and FP treatments under both irrigation methods were not significantly different. The net benefit of OMI treatments was lower than FP treatments under flood irrigation. Comparatively, the net benefit of OMI treatments was slightly higher than that of FP treatments under drip irrigation. Additionally, the net benefit of the drip system was slightly lower than that of the flood irrigated treatment due to the cost of drip tapes.

**Table 3:** The costs and benefits of input and output materials for maize production.

Treatment	N	P_2_O_5_	K_2_O	Drip tape	Irrigation	Others	Yield benefit	Net benefit
Flood irrigation								
Control	0.00 (0.00)	0.24 (1.00)	0.54 (2.28)	0.00 (0.00)	0.40 (1.70)	4.47 (18.93)	10.56 (44.68)	4.91 (20.76)
OM	0.68 (2.89)	0.24 (1.00)	0.54 (2.28)	0.00 (0.00)	0.40 (1.70)	4.47 (18.93)	19.41 (82.14)	13.08 (55.34)
OMI	0.92 (3.89)	0.24 (1.00)	0.54 (2.28)	0.00 (0.00)	0.40 (1.70)	4.47 (18.93)	21.69 (91.78)	15.12 (63.98)
FP	1.52 (6.44)	0.24 (1.00)	0.54 (2.28)	0.00 (0.00)	0.40 (1.70)	4.47 (18.93)	23.63 (100.0)	16.46 (69.65)
Drip irrigation								
Control	0.00 (0.00)	0.20 (0.86)	0.44 (1.88)	1.73 (7.30)	0.28 (1.18)	4.47 (18.93)	12.57 (53.19)	5.57 (23.58)
OM	0.63 (2.65)	0.20 (0.86)	0.44 (1.88)	1.73 (7.30)	0.28 (1.18)	4.47 (18.93)	21.22 (89.79)	13.6 (57.53)
OMI	0.92 (3.89)	0.20 (0.86)	0.44 (1.88)	1.73 (7.30)	0.28 (1.18)	4.47 (18.93)	22.04 (93.26)	14.12 (57.76)
FP	1.39 (5.89)	0.20 (0.86)	0.44 (1.88)	1.73 (7.30)	0.28 (1.18)	4.47 (18.93)	22.02 (93.19)	13.64 (57.70)

The unit of costs and benefits is 1,000 yuan ha^−1^. The relative value indicator is the yield benefit of FP treatment at flood irrigation as 100% and provides all other figures [the yield benefit of FP treatment should appear as 23.63 (100.0) and the N fertilizer of OM treatment as 0.68 (2.89)].

### 3.2 Nr losses and N footprint

**Figure 4:**
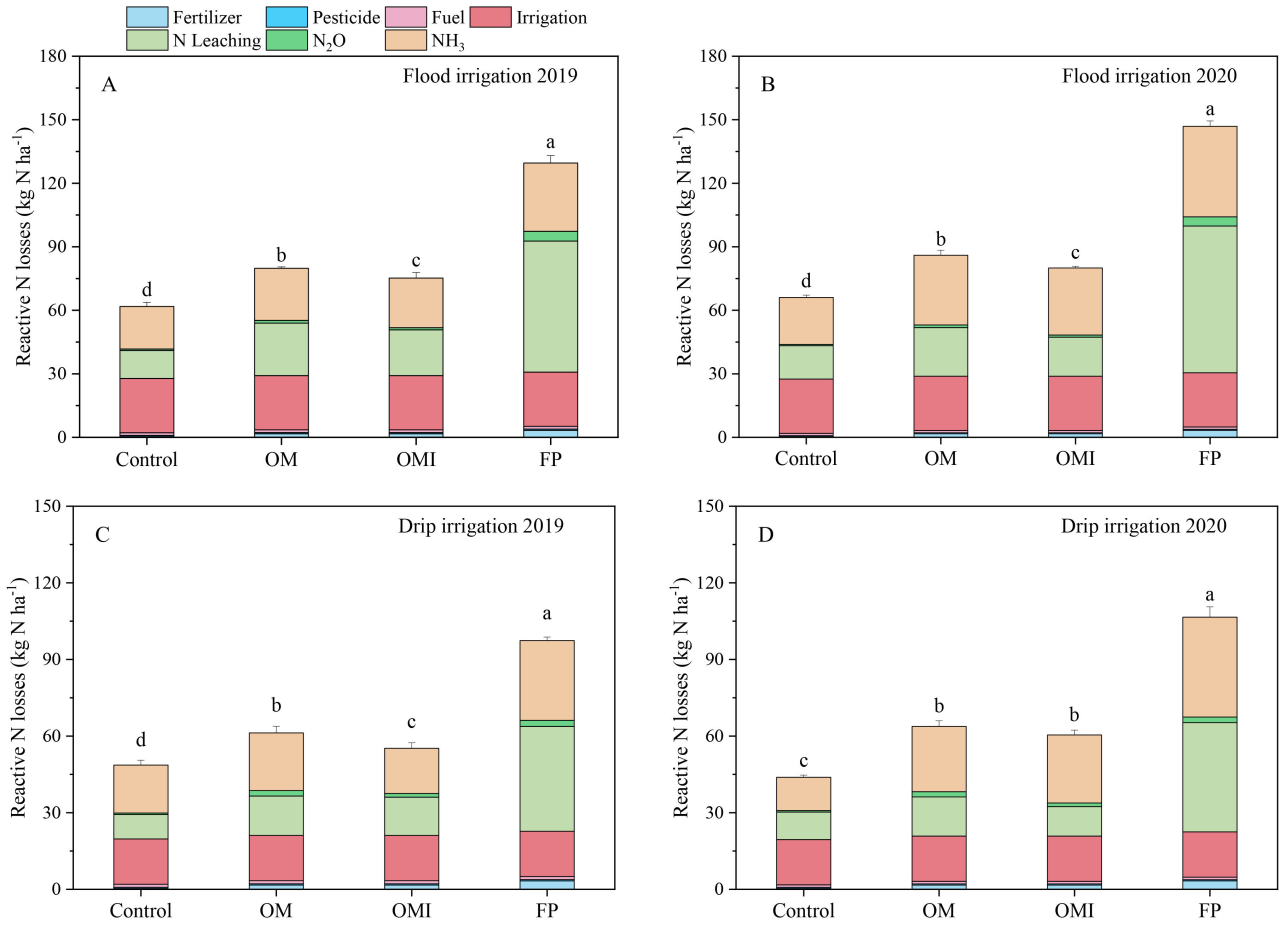
Reactive N losses in flood and drip irrigation of maize system. Panels **(A, B)** represent the annual Nr losses of the flood irrigation system. Panels **(C, D)** represent the Nr losses of the drip irrigation system. Different lowercase letters indicate significant differences at *p*< 0.05 (LSD).

**Figure 5:**
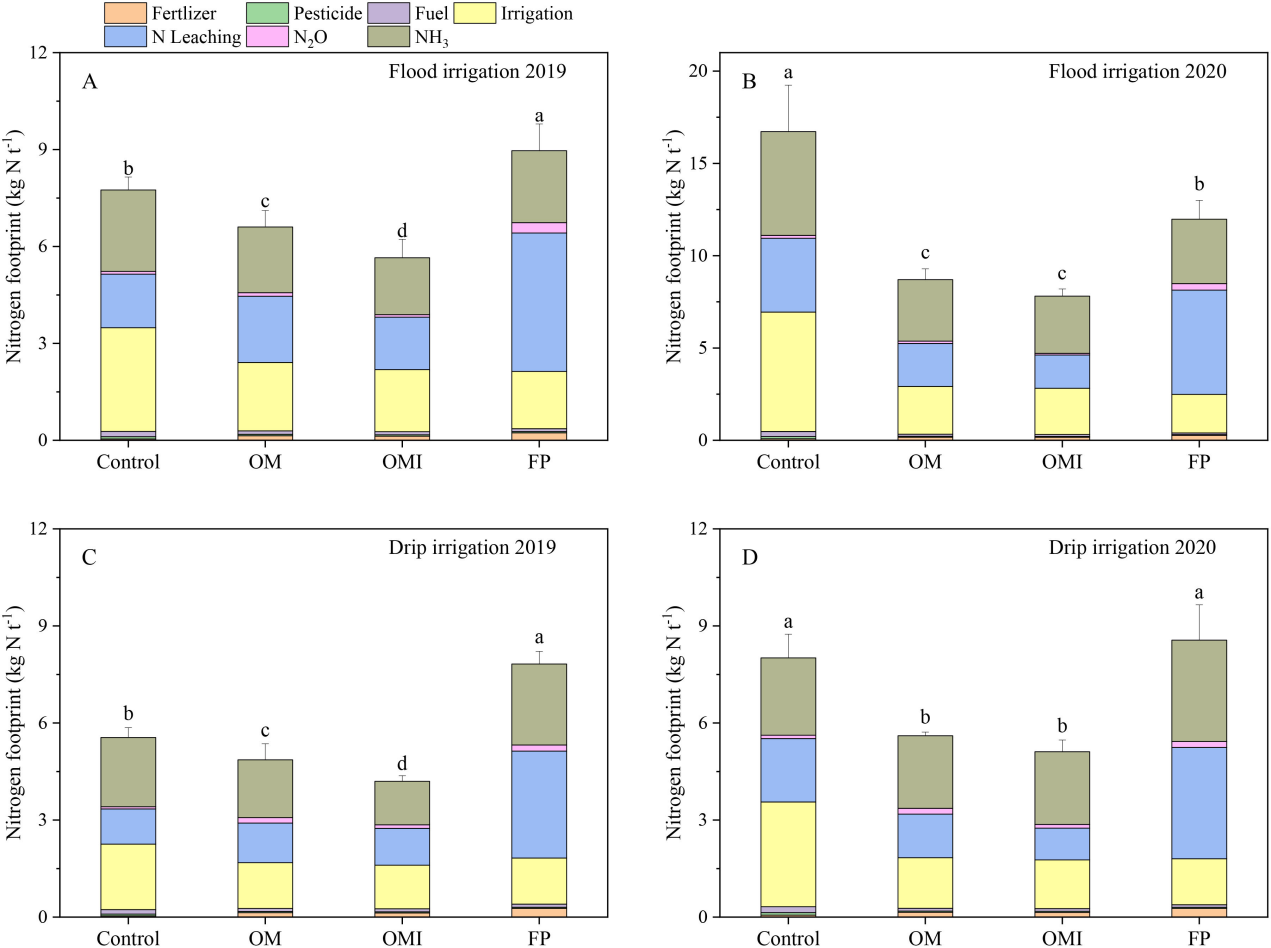
Nitrogen footprint in flood and drip irrigation of maize system. Panels **(A, B)** represent the annual NF of the flood irrigation system. Panels **(C, D)** represent the NF of the drip irrigation system. Different lowercase letters indicate significant differences at *p*< 0.05 (LSD).

The electricity for irrigation, N leaching, and NH_3_–N volatilization were the prime contributors to the Nr losses and NF in flood and drip irrigated maize systems (**Figures 4**, **5**). The N leaching and NH_3_–N volatilization of the FP treatment were significantly higher than those of other treatments. **Figures 4A, B** show that the Nr losses from OM, OMI, and FP treatments were 79.9 kg N ha^−1^, 75.2 kg N ha^−1^, and 129.6 kg N ha^−1^ in 2019 and 86.0 kg N ha^−1^, 80.0 kg N ha^−1^, and 146.9 kg N ha^−1^ in 2020 under flood irrigation, respectively. The Nr loss amount of various N treatments in 2020 was slightly higher than in 2019. Compared with FP treatment, the Nr losses of OM and OMI treatments were significantly reduced by 38.4% and 41.9% in 2019 and 41.5% and 45.5% in 2020, respectively. In addition, the Nr losses of OMI treatment were 5.8% and 7.0% lower than those of the OM treatment in 2019 and 2020, respectively. Under drip irrigation (**Figures 4C, D**), the Nr losses of OM, OMI, and FP treatments were 61.2 kg N ha^−1^, 55.2 kg N ha^−1^, and 97.4 kg N ha^−1^ in 2019 and 63.8 kg N ha^−1^, 60.5 kg N ha^−1^, and 106.6 kg N ha^−1^ in 2020, respectively. The Nr losses of the OM and OMI treatments were notably lower than those of the FP treatment, which were reduced by 37.1% and 43.3% in 2019 and 40.2% and 43.3% in 2020, respectively. The Nr losses of OMI treatment were less than 9.8% in 2019 and 5.2% in 2020 of the OM treatment, respectively.

The NF of OM and OMI was lower than the FP treatment (**Figure 5**) without compromising the grain yield (**Figure 3**). The NF of drip irrigation in the different N treatments was lower than that of flood irrigation. The highest NF of different treatments in 2019 and 2020 was recorded in flood irrigation, which was FP treatment (9.0 kg N t^−1^) in 2019 and control treatment (16.7 kg N t^−1^) in 2020 (**Figures 5A, B**). However, the highest NF of drip irrigation was recorded in FP treatment of 2019 and 2020 field experiments (**Figures 5C, D**, 7.8 kg N t^−1^ in 2019 and 8.6 kg N t^−1^ in 2020). Under flood-irrigated experiments, the NF of OM and OMI treatments were significantly reduced by 26.4% and 37.0% in 2019 and 27.3% and 34.8% in 2020 compared to the FP treatment, respectively. The NF of OMI treatment was 14.4% and 10.3% lower than those of the OM treatment in 2019 and 2020, respectively. The NF of the OM and OMI treatments was remarkably lower than that of the FP treatment.

### 3.3 Greenhouse gas emissions and C footprint

**Figure 6:**
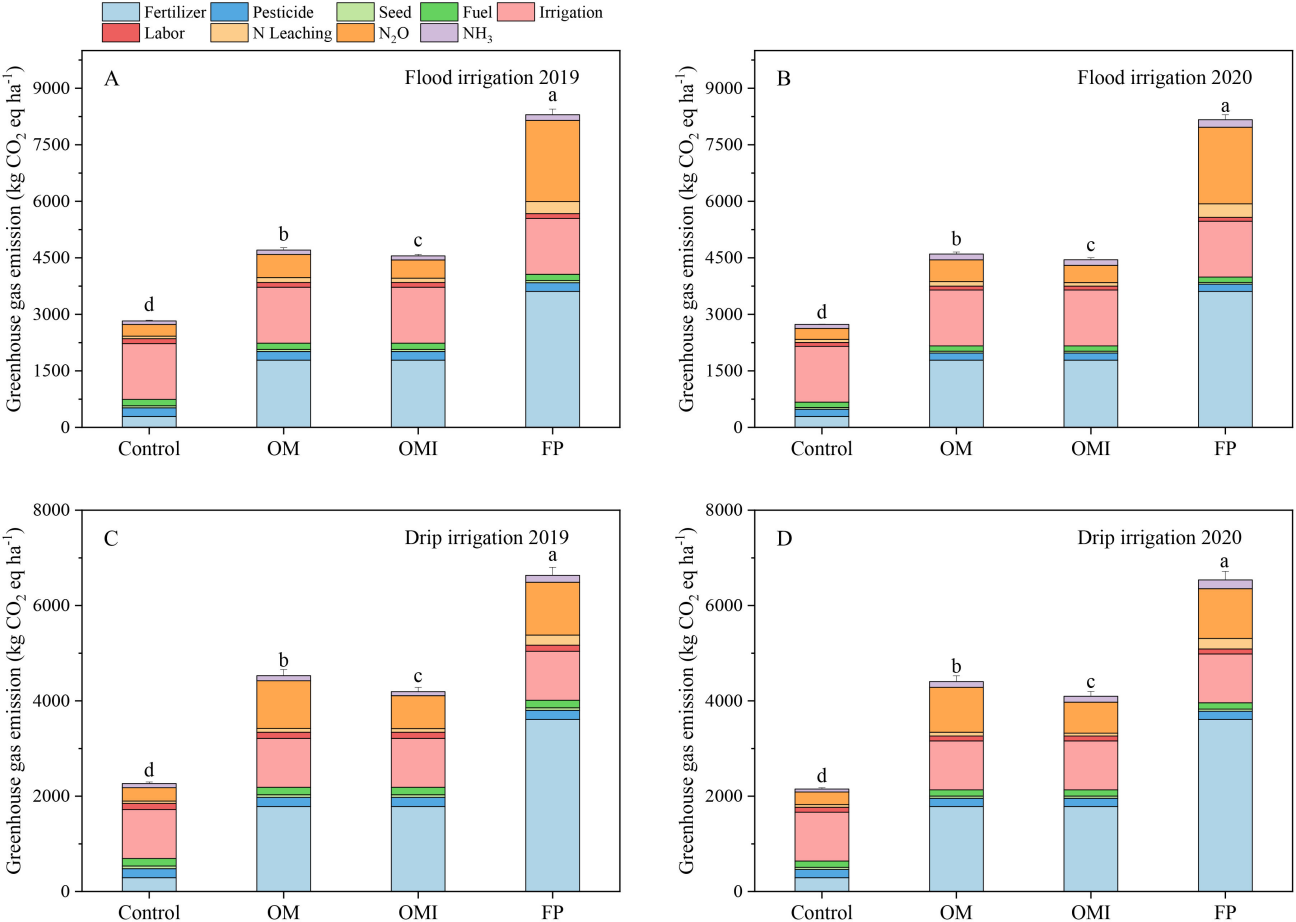
Greenhouse gas emissions in flood and drip irrigation of maize system. Panels **(A, B)** represent the annual GHG emissions of the flood irrigation system. Panels **(C, D)** represent the GHG emissions of the drip irrigation system. Different lowercase letters indicate significant differences at *p*< 0.05 (LSD).

**Figure 7:**
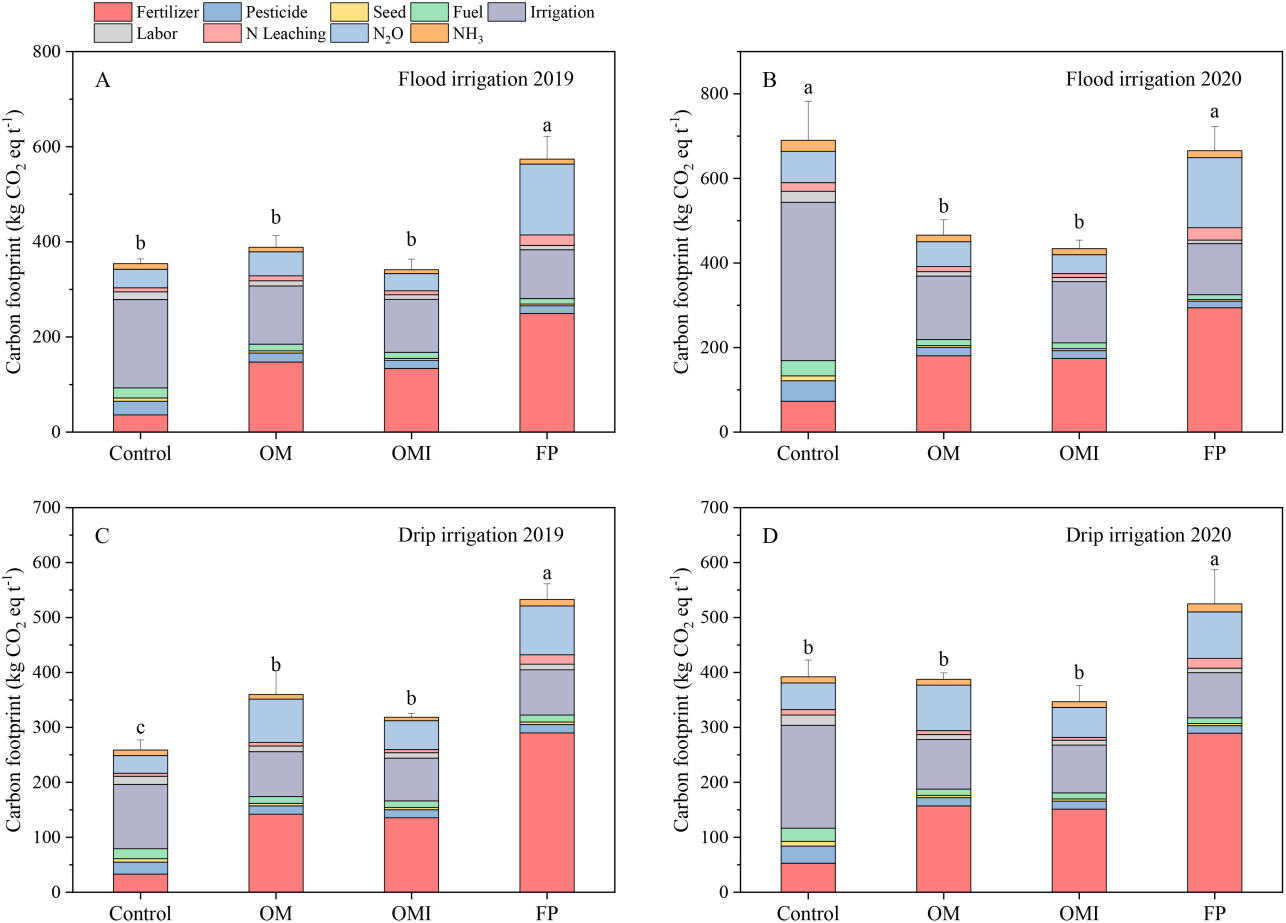
Carbon footprint in flood and drip irrigation of maize system. Note: Panels **(A, B)** represent the annual CF of the flood irrigation system. Panels **(C, D)** represent the CF of the drip irrigation system. Different lowercase letters indicate significant differences at *p*< 0.05 (LSD).

The GHG emissions and CF were significantly affected by irrigation methods and N management containing urease inhibitors to reduce environmental impacts (**Figures 6**, **7**). The GHG emissions of the FP treatment were markedly higher than those in the OM and OMI treatments (**Figure 6**). The GHG emissions of OM, OMI, and FP treatments were 4,704 kg CO_2_ eq ha^−1^, 4,552 kg CO_2_ eq ha^−1^, and 8,298 kg CO_2_ eq ha^−1^ in 2019 and 4,600 kg CO_2_ eq ha^−1^, 4,448 kg CO_2_ eq ha^−1^, and 8,164 kg CO_2_ eq ha^−1^ in 2020 under flood irrigation, respectively. The GHG emissions under drip irrigation were slightly lower than those of flood irrigation. The OM and OMI treatments were also significantly different in terms of GHG and CF. The GHG emissions of the OMI treatment were 45.1% and 3.2% lower in 2019 and 45.5% and 3.3% lower in 2020 compared to those of the FP and OM treatments, respectively. The GHG of OM treatment was 43.3% in 2019 and 43.7% lower than the FP treatment. Meanwhile, fertilizers, electricity for irrigation, and N_2_O–N emissions were the dominant components of the CF under both flood and drip irrigation in the maize systems (**Figure 7**). The CF among different N treatments in maize systems under food and drip irrigation presented similar trend to NF. The highest CF was recorded in the FP treatment and the control treatment of 2019 and 2020 under flood irrigation, and the highest CF of the drip irrigation was only recorded in the FP treatment. The CF of OM and OMI treatments were significantly reduced by 32.3% and 40.5% in 2019 and 30.0% and 34.8% in 2020 compared to the FP treatment in flood irrigation, respectively. The CF of the OMI treatment also documented a striking effect more than the OM treatment due to UI application. The CF of flood and drip irrigation presented similar outcomes and trends among the OM, OMI, and FP treatments. Compared to the FP treatment, the CF of OM and OMI treatments were significantly reduced in 2019 and 2020 under drip irrigation.

## 4 Discussion

### 4.1 Effects of N and irrigation management on grain yields

In this study, the N application rate of OMI treatment was significantly reduced while maintaining maize grain yield under flood and drip irrigation compared to the FP treatment. Drip irrigation consumed less water than flood irrigation. The principal advantages of the OMI treatment were reflected in the decent input rate of N fertilizer containing UI and irrigation methods to match maize N and water demands. The appropriate N fertilizer rates based on improved N management practices could increase crop yields with lower inevitable N losses to the environment (Cai et al., 2023; Yin et al., 2021). Our results showed that the grain yield of OM treatment was significantly lower than those of OMI and FP treatment under flood irrigation. In contrast, the maize grain yields of OM, OMI, and FP treatments presented no significant difference under drip irrigation (**Figures 3C, D**). Both N fertilizer and irrigation method were vital factors for grain yields, indicating that N and water inputs must be optimized simultaneously in crop cultivation (Du et al., 2018). However, flood irrigation systems and broadcasting of N fertilizer on the soil surface resulted in water losses by evaporation and large nutrient flow to the environment via leaching and runoff. The UI application resulted in lesser N losses to the environment and more N utilized by crops under drip irrigation than flood irrigation (Silber et al., 2003). Thus, applying N fertilizer containing UI provides a perspective to increase NUE and reduce N losses in maize systems under flood irrigation in a water-limited region of Northwest China. Drip irrigation reduced N loss and water consumption and increased N uptake, biomass accumulation, and crop yield compared to flood irrigation (Chen et al., 2020; Guo et al., 2021). Drip irrigation has excellent advantages in fertilizer input and water conservation, which can maintain favorable moisture conditions in the roots zone and facilitate the movement of nutrients to roots via diffusion. The frequencies of fertilizer and drip irrigation improve NUE and water efficiency in cropping systems (Carrijo et al., 2017; Wang et al., 2019). Drip irrigation could also enhance crop nutrient availability and uptake by synchronizing the water and nutrient supply (Si et al., 2020; Singandhupe et al., 2003).

### 4.2 Effects of N and irrigation management on N footprint

Nitrogen footprint is mainly affected by fertilizer application, electricity for irrigation, pesticide use, fuel for machinery, labor for planting, N losses from N leaching, NH_3_–N volatilization, and N_2_O–N emissions (Liang et al., 2018). In our study, the NF was calculated by the agriculture system input and output indicators per ton of standard yield in agricultural production based on LCA, which could be used to assess ecosystem sustainability (Brentrup et al., 2004; Liang et al., 2018). Several studies indicated that the NF could reach 9.1 kg N t^−1^ in the summer maize system with Nutrients Experts in Northcentral and Northeast China, 15.8 kg N t^−1^ in the USA, and 6.3 kg N t^−1^ in the EU (He et al., 2021; Huang et al., 2021; Leach et al., 2016). Our study showed that the NF of OM and OMI treatments under flood irrigation was similar to those reported in the EU but much lower than that in other parts of China and the USA. Our results showed that the NF of OM and OMI treatments under drip irrigation were significantly lower than those of other studies (He et al., 2021; Huang et al., 2021). Drip irrigation is an effective technology that can help optimize N management and improve the utilization of water resources compared to flood irrigation. Previous studies confirm the results of our finding that the electricity for irrigation, N leaching, and NH_3_–N volatilization were dominant contributors to the Nr losses and NF of maize systems under flood and drip irrigation. The other indicators of Nr losses and NF only accounted for a small percentage (**Figures 4**, **5**). The N fertilizer application rate is a critical indicator for influencing the N losses from N leaching, N_2_O–N emissions, NH_3_–N volatilization, and the NF in crop production under flood irrigation (Huang et al., 2021, 2017). Inefficient irrigation regimes are another essential indicator to control the NF of intensive irrigated agricultural systems (Nemecek et al., 2011; Wang et al., 2017). However, multiple fertilization and irrigation applications were the primary reasons for ample Nr-Mi of the NF in maize systems under drip irrigation compared to other crop systems (Grassini and Cassman, 2012; Huang et al., 2021). The average Nr losses and NF of different N treatments in drip irrigation were 24.6%–27.7% and 21.8%–44.6% lower than in flood irrigation. The average Nr losses and NF of the OMI treatment were significantly lower than those of the OM and FP treatments (**Figures 4**, **5**). The appropriate N fertilizer rates and intelligent drip irrigation regimes were necessary to reduce higher NF in maize production regions (Han et al., 2023; Xu et al., 2020). Therefore, integrating N and drip irrigation management measures to optimize agricultural production has excellent potential for minimizing environmental impacts (Król-Badziak et al., 2020; Maltese et al., 2024; Zhang et al., 2020).

### 4.3 Effects of N and irrigation management on C footprint

Carbon footprint is calculated based on fertilizers input, electricity for irrigation, pesticides, seed, fuel for machinery, labor for planting, N losses from N leaching, NH_3_–N volatilization, and N_2_O–N emissions per ton grain yield (Huang et al., 2021; Liang et al., 2018). In the present study, the GHG emissions and CF of drip irrigation were significantly reduced compared to flood irrigation. Studies have revealed that the CF in maize systems with optimized N management under flood irrigation is 436 kg CO_2_ eq t^−1^ in Northcentral Northeast China, 261 kg CO_2_ eq t^−1^ in India, and 231 kg CO_2_ eq t^−1^ in the USA (Grassini and Cassman, 2012; Huang et al., 2021; Lenka et al., 2017). In the present study, the CF of OM treatment under flood irrigation was similar to those reported in China, and the CF of OMI was higher than that in the USA and India. Comparatively, the CF of OM and OMI treatments under drip irrigation was higher than that in the USA but much lower than that of previously reported cropping systems in China. In addition, another study reported that the CF of maize under drip irrigation was significantly lower than that of rice (657 kg CO_2_ eq t^−1^) but higher than that of wheat (166 kg CO_2_ eq t^−1^) for similar yield goals (Linquist et al., 2011). Our study showed that integrating flood irrigation and UI could notably reduce N losses from N_2_O–N emissions and NH_3_–N volatilization and CF in maize systems. The UI is widely used in flood-irrigated agricultural systems to decrease gaseous N losses and increase NUE (Cheng et al., 2022). Our results also indicated that applying UI in flood irrigation significantly reduced total N losses to the environment in maize systems. Applying N-fertilizer-incorporated UI provides a practical way for farmers to reduce N overapplication and decrease environmental impacts under flood irrigation systems in arid and semi-arid regions. Better environmental performance inevitably improves the sustainability and economics of maize production. Drip irrigation performed better than flood irrigation in decreasing negative environmental impacts in crop production systems. Drip irrigation reduces the amount of N and water requirements and increases N uptake, biomass accumulation, and crop yields (Chen et al., 2020; Guo et al., 2021), which is consistent with our findings. Drip irrigation has broader application prospects in water-constrained areas to conserve natural resources and sustain crop production. Drip irrigation of different crops has been proven to reduce N rate and Nr losses compared with flood irrigation in arid and semi-arid regions (Di et al., 2024; Wang et al., 2021). Although drip irrigation has better environmental performance, adopting drip irrigation should be based on the crop production region’s climate and economic conditions.

The calculation of CF is directly affected by many driven indicators in agricultural systems based on LCA. Most relevant studies indicated that N fertilizer, N leaching, and N_2_O–N emissions are the leading indicators influencing the CF of crop production under flood or furrow irrigation (Huang et al., 2021; Zhang et al., 2021). Our study revealed that fertilizers input, electricity for irrigation, and N_2_O–N emissions were the critical factors controlling the GHG emissions and CF in flood- and drip-irrigated cropping systems. We also confirmed prior findings that the N fertilizer and electricity for irrigation are important indicators in controlling the CF of crop systems under drip irrigation (Nemecek et al., 2011; Wang et al., 2017). The N_2_O–N emission levels were also an important factor in controlling GHG emissions and CF because the N_2_O–N emission is 298-fold greater than the CO_2_ coefficient in terms of the effects on global warming (Huang et al., 2021; IPCC, 2019). Our results were consistent with previous studies that UI use significantly reduced N_2_O–N emissions under flood irrigation (Cheng et al., 2022; Elrys et al., 2021). In addition, fertilization timing and irrigation rates of drip irrigation were the primary contributors to the large GHG-Mi of the CF in maize systems under drip irrigation compared with those of other crop production models (Grassini and Cassman, 2012; Huang et al., 2021). Therefore, agricultural production should consider rational N and efficient water management to match crop requirements and regional characteristics in the future.

### 4.4 Limitations of this study

Although the integration of optimal N management and drip irrigation technology in the present study showed significant effects for improving environmental impacts, there were also some inevitable limitations. The Nr losses and GHG emissions due to agricultural material input and output results were highly dependent on the completeness and accuracy of the life cycle inventories. Our study mainly used the emission factors of life cycle inventories based on agricultural materials input and output in China, which have been widely used in earlier LCA research (Huang et al., 2021; Liang et al., 2018). However, the life cycle inventories and emission factors involve many industry and agriculture sectors, which are quite challenging to collect. Despite these limitations and uncertainties, our study considered the characteristics of environmental footprints to provide meaningful analysis for distinguishing maize production under flood and drip irrigation. Improving N management and irrigation methods can eventually achieve a win–win outcome in mitigating environmental impacts. However, this process may be slow, as the required technology is not yet widely available in China.

## 5 Conclusions

This study analyzed the C and N footprints of maize production affected by N application rates with or without urease inhibitor under flood and drip irrigation using LCA. The OMI treatment showed no significant difference in maize grain yield but greatly reduced N_2_O–N emissions and NH_3_–N volatilization and decreased C and N footprints in both flood and drip irrigation compared to the FP treatment. The Nr losses, GHG emissions, NF, and CF of the OMI and OM treatments were remarkably lower than those of the FP treatment, and the CF of OM and OMI treatments had no notable difference between flood and drip irrigation. Drip irrigation significantly reduces Nr losses, GHG emissions, NF, and CF compared to flood irrigation. The N leaching, electricity for irrigation, and NH_3_–N volatilization were the most dominant contributors to NF of maize systems, and the fertilizers (N, P_2_O_5_, and K_2_O), electricity for irrigation, and N_2_O–N emissions were the most dominant contributors to CF of maize systems. Therefore, integrating N management practices and irrigation methods would improve maize production sustainability and reduce environmental impacts. Future research should focus more on integrating N fertilizer input, efficient management approaches, minimal environmental impact, and maximum economic benefit in cropping systems.

## Data Availability

The raw data supporting the conclusions of this article will be made available by the authors, without undue reservation.
